# Homo- and Copolymerization of Ethylene with Norbornene Catalyzed by Vanadium(III) Phosphine Complexes

**DOI:** 10.3390/molecules24112088

**Published:** 2019-05-31

**Authors:** Giorgia Zanchin, Alessia Gavezzoli, Fabio Bertini, Giovanni Ricci, Giuseppe Leone

**Affiliations:** CNR-Istituto per lo Studio delle Macromolecole (ISMAC), via A. Corti 12, I-20133 Milano, Italy; giorgia.zanchin@ismac.cnr.it (G.Z.); alessia.gavezzoli@gmail.com (A.G.); fabio.bertini@ismac.cnr.it (F.B.); giovanni.ricci@ismac.cnr.it (G.R.)

**Keywords:** vanadium, ethylene/norbornene copolymerization, phosphine

## Abstract

Herein, we report the homo- and co-polymerization of ethylene (E) with norbornene (NB) catalyzed by vanadium(III) phosphine complexes of the type VCl_3_(PMe_n_Ph_3-n_)_2_ [n = 2 (**1a**), 1 (**1b**)] and VCl_3_(PR_3_)_2_ [R = phenyl (Ph, **1c**), cyclohexyl (Cy, **1d**), *tert*-butyl (*t*Bu, **1e**)]. In the presence of Et_2_AlCl and Cl_3_CCOOEt (ETA), **1a**–**1e** exhibit good activities for the polymerization of ethylene, affording linear, semicrystalline PEs with a melting temperature of approximately 130 °C. Mainly alternating copolymers with high comonomer incorporation were obtained in the E/NB copolymerization. A relationship was found between the electronic and steric properties of the phosphine ligands and the catalytic performance. Overall, the presence of electron-withdrawing ligand substituents increases the productivity, complexes with aryl phosphine (weaker σ–donor character) exhibiting a higher (co)polymerization initiation rate than those with alkyl phosphines (stronger σ–donor character). Steric effects also seem to play a key role since **1d** and **1e**, having large size phosphines (PCy_3_ θ = 170° and P*t*Bu_3_ θ = 182°, respectively) are more active than **1a** (PMe_2_Ph θ = 122°). In this case, the larger size of P*t*Bu_3_ and PCy_3_ likely compensates for their higher donor strength compared to PMe_2_Ph.

## 1. Introduction

Phosphines are one of the most important families of ligands in organometallic chemistry [1,2]. The synthesis, structure and reactivity of transition metal phosphine complexes continue to attract considerable attention due to their ability to undergo different transformations in which the phosphine is involved in the reactivity or acts as spectator ligand [3,4,5]. Phosphine ligands are typically constructed with combinations of alkyl and aryl groups, and both steric and electronic properties of the ligand can be modulated for specific applications. In addition, the relatively low cost and large variety of phosphines are incentives for the development of metal–phosphine complexes.

Vanadium is an element of growing importance. With multiple oxidation states and coordination number and flexible geometry, applications of vanadium complexes in catalysis and polymer science continue to grow [6,7,8,9,10]. Today, vanadium catalysts are largely used for the production of elastomers [11], high-molecular-weight poly(ethylene)s (PEs) with narrow molecular weight distribution [12,13], syndiotactic poly(propylene)s [14,15], and ethylene copolymers with cyclic olefins (COCs) [16,17] and α-olefins [18,19]. Among them, there is an increased interest in COCs because of the easy availability of the monomers and unique copolymer properties. The most versatile and interesting COCs are the copolymers of ethylene (E) with norbornene (NB), which have gained much attention as high-tech engineering plastics [20,21]. A significant breakthrough in the field of COCs was achieved in 1991 when Kaminsky discovered that C2-symmetric Group 4 metallocenes, in combination with methylalumoxane (MAO), catalyzed the copolymerization of ethylene with cyclic olefins [22]. This discovery also stimulated the development of post-metallocenes with early and late transition metals, expanding the commercially useful transition metals, but also broadening the range of COCs accessible by insertion polymerization [23].

For the reasons mentioned above concerning the versatility of vanadium complexes and the increasing relevance of COCs, in recent years, we became interested in the synthesis of vanadium complexes, and their application in the polymerization of various (di)olefins [16,17,24]. In this framework, recently, we reported that VCl_3_(PMePh_2_)_2_, in combination with Et_2_AlCl and ETA, catalyzed the E/NB copolymerization [24]. Mainly alternating copolymers, in the presence of a low excess of Et_2_AlCl and ETA, were successfully obtained. Vanadium–phosphine complexes have been known since the mid 1980s by the pioneer work of Caulton and Larkworthy [25,26,27,28], but have been less investigated in the context of olefin (co)polymerization with respect to vanadium complexes bearing multidentate chelating and anionic ligands having, in different combination, N and O hard donor atoms [29,30,31,32,33,34]. In this paper, we report the homo- and copolymerization of ethylene with NB catalyzed by a series of vanadium(III) complexes with tertiary phosphine of the type PMe_n_Ph_3-n_ with n = 2 (**1a**), n = 1 (**1b**), and PR_3_ with R = Ph (**1c**), Cy (**1d**), *t*Bu (**1e**). We found that a relationship exists between the electronic and steric properties of the phosphine ligand and the catalytic performance, and that the substituents on the phosphorus atom strongly affect the molecular weight of the resulting (co)polymers. The homo- and copolymerization of ethylene with NB using VCl_3_(THF)_3_ under the same set of conditions were performed as well for comparison.

## 2. Results and Discussion

### 2.1. Polymerization of Ethylene

First, **1a**–**1e** were screened for the polymerization of ethylene under the same set of conditions of 1 atm of ethylene, at 20 °C, in toluene. Et_2_AlCl was selected as co-catalyst due to exhibiting higher activities than MAO [24]. Indeed, the V–alkyl species formed by some vanadium complexes and Et_2_AlCl were found to be in equilibrium between chloro-bridged and cationic alkyl species due to the smaller bulkiness and stronger nucleophilic nature of Et_2_AlCl [6,10], while an isolated cationic species would be formed in the presence of MAO [10,29]. The polymerization experiments were carried out in the presence of ETA (50 equiv. to V) to overcome the main disadvantage of using vanadium(III) complexes, which is the rapid deactivation associated with the reduction to inactive vanadium(II) species. The results are listed in Table 1, and are presented as a function of the phosphine donor ability [as measured by the electronic parameter υ_CO_ based on the carbonyl stretching frequencies in Ni(CO)_3_L complexes (L = phosphine ligand)] and steric properties (measured by cone angle, θ) as defined by Tolman [35]. Table 1 also reports the results for the polymerization of ethylene by VCl_3_(THF)_3_ employed in this study for comparison. 

**Figure 1:**
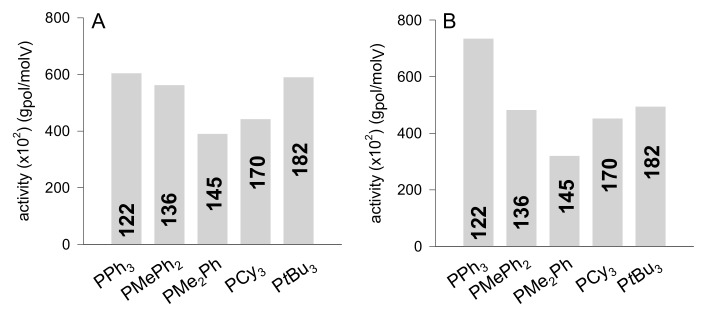
Plot of activity, calculated as g_pol_/mol_V_ vs. the vanadium complexes employed [PPh_3_ (**1c**), PMePh_2_ (**1b**), PMe_2_Ph (**1a**), PCy_3_ (**1d**) and P*t*Bu_3_ (**1e**)] for (**A**) the polymerization of ethylene and (**B**) the copolymerization of ethylene with norbornene (NB). In each bar, the phosphine cone angle (°) is reported.

All the vanadium phosphine complexes exhibited good activities for the polymerization of ethylene, (Figure 1A) affording linear, semicrystalline PEs with a melting temperature of approximately 130 °C (Figure S1 and S2 in Supplementary Materials). The activities of the vanadium phosphine complexes were lower than VCl_3_(THF)_3_, likely due to the faster displacement of the THF ligand (indeed THF is expected to be a weak donor ligand and thus to dissociate faster), but also due to different ratios of the dissociated ligand re-insertion rate relative to the ethylene insertion/coordination rate [24].

Generally, both the activity and the molecular weight of the resulting PEs were strongly affected by the substituents on the phosphorus atom, although the relationship between the catalytic activity, the polymer molecular weight and the phosphine ligand is not straightforward. This is because phosphine ligands are involved in dynamic “on/off” events, which include dissociative and associative pathways, and competitive reactions [24], and electronic and steric effects are intimately connected and difficult to separate [36,37,38,39].

Changing the substituents on the phosphorus atoms yielded large changes in the ligand donor properties: aryl phosphines (weaker σ-donor character) being weakly coordinated to the metal compared to alkyl phosphines (stronger σ-donor character). However, according to the parameters defined by Tolman [35], a general trend seems to emerge from data in Table 1. For complexes having at least one phenyl group on the phosphorus atoms, i.e., VCl_3_(PMe_n_Ph_3-n_)_2_ and VCl_3_(PPh_3_)_2_, the activity increased in the order **1a** (PMe_2_Ph) < **1b** (PMePh_2_) ≤ **1c** (PPh_3_) with decreasing ligand donor strength (Table 1 and Figure 1A). Steric effects also play a role. In fact, **1e** (PtBu_3_, the stronger σ-donor, θ = 182°) gave an activity close to that of **1b** (PMePh_2_, θ = 136°): in this case, the bigger size of **1e** compared to **1b** more than compensates for its higher donor strength (Figure 1A). Moreover, the effect of the steric perturbation is better illustrated by the differences in activity between **1d** (PCy_3_) and **1e** (PtBu_3_). These two complexes have similar electronic properties (**1d** υ = 2056.1 cm*^−^*^1^; **1e** υ = 2056.4 cm*^−^*^1^), but **1e** exhibited an activity higher than **1d** likely due to the larger cone angle of PtBu_3_ (θ = 182°) compared to that of PCy_3_ (θ = 170°) (Figure 1A).

PEs obtained from the vanadium phosphine complexes had higher molecular weight than the polymer obtained from VCl_3_(THF)_3_, and in some cases even two times more. This is likely because VCl_3_(THF)_3_ is more prone to β–H elimination, followed by a fast displacement of a vinyl-terminated polymer, than the vanadium phosphine complexes. Complexes containing phosphines with an increased σ-donor character gave PEs with higher molecular weight: **1e** (P*t*Bu_3_, the strongest σ-donor) gave the polymer with the highest molecular weight (Table 1, entry 5 *M*_w_ = 84,300 g/mol), while PPh_3_ (the weakest σ-donor) gave the polymer with the lowest molecular weight (Table 1, entry 3 *M*_w_ = 35,300 g/mol), close to that obtained from VCl_3_(THF)_3_ (Table 1, entry 6 *M*_w_ = 34,800 g/mol). It is worthwhile to note that the molecular weight of PEs obtained from complexes having aryl phosphines increased in the reverse order with respect to the activity; that is **1c** (PPh_3_, *M*_w_ = 35,300 g/mol) < **1b** (PMePh_2_, 44,600) < **1a** (PMe_2_Ph, 65,500). 

### 2.2. Copolymerization of Ethylene with Norbornene

Then, **1a**–**1e** were used for the E/NB copolymerization under the same set of conditions of 1 atm of ethylene, at 20 °C, in toluene, with Et_2_AlCl (500 equiv to V), ETA (50 equiv to V) and for a NB/E feed ratio of 4. The copolymerizations were performed at short reaction times to ensure the uniformity of the catalytic process, even in the presence of a very high comonomer consumption in the early instants of the reaction, and thus to obtain copolymers with uniform composition [16,17,24]. The results are summarized in Table 2. 

None of the investigated complexes was active in the homopolymerization of NB under the conditions employed, while high activities were obtained for the copolymerization of E/NB in the first seconds of the reaction. When both the comonomers are available for the active intermediates, all the complexes are instantaneously activated and the copolymerization proceeds at significant rates, particularly at the initial stage. The increased activity could be due to the coordination of the nucleophilic and sterically demanding NB that, in some cases, is expected to stabilize the active site [40].

In general, we found the same trend in activity observed for the homopolymerization of ethylene, complexes with aryl phosphines being more active than those with alkyl phosphines (Figure 1B vs. Figure 1A). For complexes having at least one phenyl group on the phosphorus atoms, i.e., **1a**–**1c**, the activity increased in the order **1a** (PMe_2_Ph) < **1b** (PMePh_2_) < **1c** (PPh_3_), **1c** being the most active according to the lower donor strength of PPh_3_ (Figure 1B). Moving to complexes bearing alkyl phosphines, i.e., **1d** and **1e**, the activity increased with respect to **1a,** likely due to the larger cone angle of P*t*Bu_3_ and PCy_3_ (θ = 182 and 170°, respectively) despite the higher donor strength of P*t*Bu_3_ and PCy_3_ compared to PMe_2_Ph (θ = 145°). In this case, the bigger size of **1d** and **1e** compared to **1b** more than compensates for their higher donor strength (Figure 1B). Also, of note, moving from **1d** to **1e,** the activity did not increase to the same extent as found for the homopolymerization of ethylene (Figure 1A vs. Figure 1B). This may be likely due to the increased steric interaction between the bulky NB and the bulky *tert*-butyl substituents on the phosphorous atoms in **1e**, which would offer a significant impediment to the coordination and insertion of the bulkier comonomer, thus depressing propagation rate.

As observed in the case of ethylene polymerization, the average molecular weight of the copolymers generally increased with increasing the ligand σ-donor character, in the order **1c** < **1b** < **1a** ≈ **1d** < **1e**, **1c** (PPh_3_) giving the copolymer with the lowest molecular weight (*M*_w_ = 42,600 g/mol) close to that obtained with VCl_3_(THF)_3_ (*M*_w_ = 43,600 g/mol). Overall, the increase in the ligand σ–donor ability has a positive effect on the (co)polymer molecular weight: a stronger electron-donating ligand may stabilize the electron-deficient vanadium intermediate and reduce the rate of β–H elimination and subsequent chain-transfer [17,41]. Conversely, electron-withdrawing ligand substituents reduce the electron density at the metal center, resulting in higher rates of β–H elimination, but significantly enhancing catalytic performance. This could be explained assuming that β–H elimination is the rate-determining step. Since the cyclic olefin prevents β–H elimination because of Bredt’s rule, the molecular weight of the copolymers is generally higher than that of PEs.

On the other hand, the type of phosphine ligand had no significant effect on the NB incorporation and copolymer microstructure. All the copolymers have a NB content ranging from 37.8 to 39.3 mol%, regardless of the steric and electronic properties of the phosphine ligand. The same result was found by some of us [16,17], and by Yue-Sheng Li using V(III) complexes bearing β-enaminoketonato ligands [33] and different tridentate salicyladiminato ligands [34].

**Figure 2:**
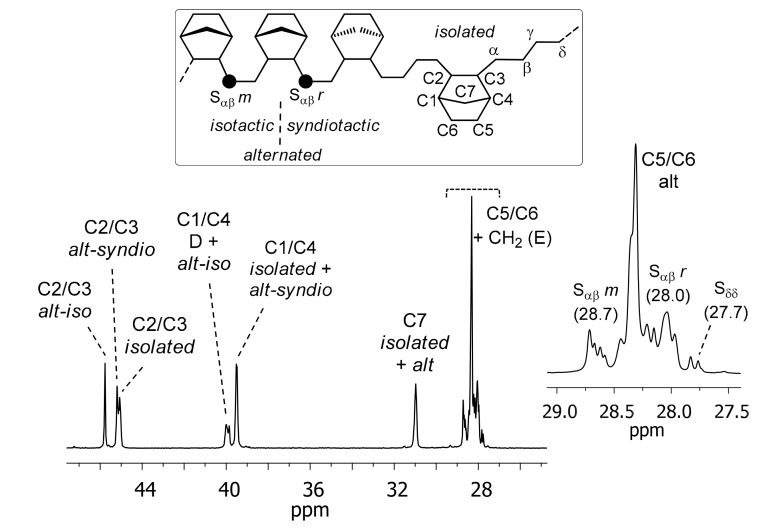
^13^C-NMR spectrum of entry 8 (Table 2, NB = 37.8 mol%).

The copolymer microstructure was investigated by ^13^C-NMR. The signals of each chemical shift region were assigned, according to the literature [42], as follows: 42.0–54.0 ppm, C2/C3; 34.5–42.0 ppm, C1/C4; 31.0–34.5 ppm, C7; 26.0–31.0 ppm, C5/C6 and ethylene CH_2_ (Figure 2). The ^13^C-NMR spectra of all the copolymers show the typical pattern of alternating copolymers from an addition-type copolymerization of NB with *cis exo*-*exo* enchainment. The dominant signals are those of isolated and alternating NB units, with traces of diads. As an example, the ^13^C-NMR spectrum of entry 8 (Table 2, NB = 37.8 mol%) is shown in Figure 2.

The relative intensities of peaks at 45.8 and 45.2 ppm, assigned to C2/C3 of NB in the alternating isotactic and syndiotactic NB–E–NB–E–NB sequences, respectively, also revealed that the copolymers have a random tacticity, the ratio of isotactic and syndiotactic units being in the range from 35:32 to 28:33.

**Figure 3:**
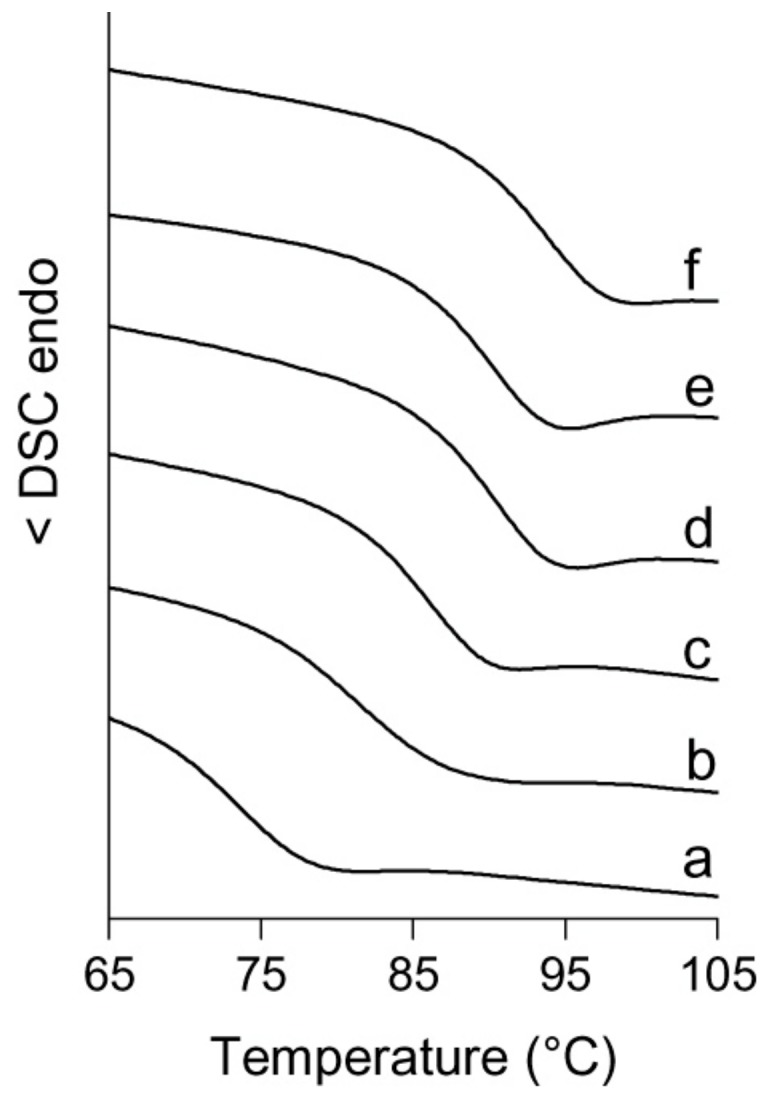
DSC curves (second heating) of E/NB copolymers obtained with (a) VCl_3_(THF)_3_ (entry 12, NB = 41.2 mol%), (b) **1c** (entry 9, NB = 38.7 mol%), (c) **1e** (entry 11, NB = 38.8 mol%), (d) **1a** (entry 7, NB = 38.0 mol%), (e) **1b** (entry 8, NB = 37.8 mol%) and (f) **1d** (entry 10, NB = 39.3 mol%).

The analysis of copolymer thermal properties revealed no melting events, but only a unique glass transition temperature (*T*_g_) in the range from 73 to 93 °C, thus suggesting that the copolymers are amorphous possibly with a homogeneous composition [43,44]. It is expected that for random copolymers with comparable *M*_w_, *T*_g_ increases with increasing NB incorporation in the copolymers [45,46]. However, as it can be seen in Table 2 and Figure 3, the copolymer generated with VCl_3_(THF)_3_ (sample 12, *M*_w_ = 43,600 g/mol), despite the highest content of NB (41.2 mol%), has the lowest *T*_g_ (73 °C) even when compared with sample 9 of the same molecular weight (*M*_w_ = 42,600 g/mol, *T*_g_ = 81 °C). We attribute this result to the fact that sample 12 is probably less homogenous in terms of composition distribution, likely due to a faster initiation efficiency of VCl_3_(THF)_3_ and hence to a more pronounced compositional drift. This leads to the formation of NB-rich copolymer chains at the beginning of the copolymerization and E-rich chains at the end, causing a non-homogeneous distribution of the comonomer that helps lower the *T*_g_ [17,24].

## 3. Materials and Methods

### 3.1. General Procedures and Materials

The manipulation of air- and/or moisture-sensitive reagents was performed under an inert atmosphere using a dual vacuum/nitrogen line and standard Schlenk-line techniques. Ethylene and nitrogen were purified by passage over columns of CaCl_2_ and molecular sieves. Oxygen was removed by fluxing the gases through BTS catalysts. Toluene (Aldrich, St. Louis, MO, USA, 99.5%) was refluxed over Na for 8 h and then distilled and stored over molecular sieves. ETA (Aldrich, 97%) was stirred over CaH_2_ for 4 h and then distilled trap-to-trap. Et_2_AlCl (Aldrich), and VCl_3_(THF)_3_ (Aldrich) were used as received without further purification. NB (Aldrich, 99%) was stirred over molten potassium at 80 °C under nitrogen for 4 h and then distilled. A stock solution was prepared by dissolving 50 g of freshly distilled NB in 86.2 mL of toluene. A deuterated solvent for NMR measurements (C_2_D_2_Cl_4_) (Aldrich, >99.5% atom D) was used as received. All the vanadium phosphine complexes were synthesized according to that already reported in the literature [47].

### 3.2. General (co)polymerization Procedure

Polymerizations were carried out in a 25-mL round-bottomed Schlenk flask. Prior to starting polymerization, the reactor was heated to 110 °C under vacuum for 1 h and backfilled with nitrogen. The reactor was charged with toluene, NB, ETA and Et_2_AlCl in that order, and then the solution was degassed, and ethylene was added until saturation. Polymerization was started by adding a toluene solution (2 mg/mL) of a vanadium complex via syringe under continuous flow of ethylene. Polymerizations were stopped with methanol containing a small amount of hydrochloric acid; the precipitated polymers were collected by filtration, repeatedly washed with fresh methanol and finally dried in vacuum at room temperature to constant weight.

### 3.3. Characterization

NMR spectra were recorded on a Bruker (Bruker Italia Srl, Milano, Italy) NMR advance 400 Spectrometer operating at 400 MHz (^1^H) and 100.58 MHz (^13^C), working in the PFT mode at 103 °C. The ^13^C experiments were performed with a 10-mm probe in C_2_D_2_Cl_4_ and referred to hexamethyldisiloxane as the internal standard. Thermal analysis was performed with a differential scanning calorimeter (DSC) Perkin Elmer 8000 (Waltham, MA, USA)). The scans were recorded in a flowing nitrogen atmosphere at scanning rates of 10 °C/min. The molecular weight average (*M*_w_) and the molecular weight distribution (*M*_w_/*M*_n_) were obtained by a high temperature Waters GPCV2000 (Milford, MA, USA) size-exclusion chromatography (SEC) system equipped with a refractometer detector. The experimental conditions consisted of three PL Gel Olexis columns, *ortho*-dichlorobenzene (*o*-DCB) as the mobile phase, a 0.8-mL/min flow rate, and 145 °C temperature. The calibration of the SEC system was constructed using eighteen narrow *M*_w_/*M*_n_ poly(styrene) standards with *M*_w_s ranging from 162 to 5.6 × 10^6^ g/mol. For SEC analysis, approximately 12 mg of polymer was dissolved in 5 mL of DCB with 0.05% of BHT as antioxidant.

## 4. Conclusions

Vanadium(III) complexes bearing commercially available monodentate phosphines exhibit good activity for the polymerization of ethylene and the E/NB copolymerization in the presence of Et_2_AlCl and ETA. Introduction of NB in the polymerization mixture accelerates the propagation rate and restrains chain termination to some extent. Alternating copolymers with high NB incorporation were produced within the first copolymerization seconds so that copolymers with a homogeneous composition were obtained only by keeping a short reaction time to limit the undesired compositional drift.

A relationship was found to exist between the phosphine electronic and steric properties and the catalytic performance. Overall, electron-withdrawing ligand substituents significantly enhance catalytic performance, vanadium complexes with aryl phosphine (weaker σ–donor character) exhibiting a higher initiation rate than those with alkyl phosphines (stronger σ–donor character). Steric properties also have a relevant effect, with complexes with great steric hindrance, such as PCy_3_ and P*t*Bu_3_ ligands, exhibiting a higher initiation rate than PMe_2_Ph (weaker σ–donor). In this case, the larger cone angle of PCy_3_ and P*t*Bu_3_ more than compensates for their higher donor strength compared to PMe_2_Ph. The type of phosphine ligand also strongly affects the (co)polymer molecular weight. Complexes with electron-donating phosphines afford (co)polymers with higher molecular weight, likely because an electron-donating ligand stabilizes the electron-deficient catalytic intermediate, reducing the rate of chain transfer.

## Figures and Tables

**Table 1:** Polymerization of ethylene catalyzed by **1a**–**1e** in combination with Et_2_AlCl.^1.^

Entry	V-cat	Phosphine			Yield (mg)	Activity ^4^ (×10 ^2^)	*M*_w_^5^ (×10 ^3^)	*M*_w_/*M*_n_^5^
		(type)	υ_CO_^2^ (cm^−1^)	*θ*^3^ (°)				
1	**1a**	PMe_2_Ph	2065.3	122	195	390	65.5	6.8
2	**1b**	PMePh_2_	2067.0	136	281	562	44.6	3.9
3	**1c**	PPh_3_	2068.9	145	302	604	35.3	4.2
4	**1d**	PCy_3_	2056.4	170	221	442	56.9	6.3
5	**1e**	P*t*Bu_3_	2056.1	182	295	590	84.2	7.9
6	VCl_3_(THF)_3_				361	722	34.7	5.0

^1^ Polymerization conditions: ethylene pressure, 1.01 bar; total volume, 25 mL (toluene); V-complex, 5 μmol; Al/V = 500; ETA/V = 50; temperature, 22 °C; time, 2 min; ^2^ measure of the electron-donating properties of phosphine ligands, as reported by Tolman [35]; ^3^ phosphine cone angle, as reported by Tolman [35]; ^4^ activity in g_pol_/mol_V_; ^5^ determined by size-exclusion chromatography (SEC).

**Table 2:** Copolymerization of E/NB catalyzed by **1a**–**1e** in combination with Et_2_AlCl and ETA.^1.^

Entry	V-cat	Phosphine			Yield (mg)	Activity ^4^ (×10 ^2^)	NB ^5^ (mol%)	*M*_w_^6^ (×10 ^3^)	*M*_w_/*M*_n_^6^	*T*_g_^7^ (°C)
		(type)	υ_CO_^2^ (cm^−1^)	*θ*^3^ (°)						
7	**1a**	PMe_2_Ph	2065.3	122	160	320	38.0	90.3	4.2	90
8	**1b**	PMePh_2_	2067.0	136	241	482	37.8	61.4	3.8	90
9	**1c**	PPh_3_	2068.9	145	367	734	38.7	42.6	4.2	81
10	**1d**	PCy_3_	2056.4	170	226	452	39.3	86.4	5.9	93
11	**1e**	P*t*Bu_3_	2056.1	182	247	494	38.8	130.0	7.0	85
12	VCl_3_(THF)_3_				395	790	41.2	43.6	4.4	73

^1^ Polymerization conditions: ethylene pressure, 1.01 bar; total volume, 25 mL (toluene); V-complex, 5 μmol; Al/V = 500; ETA/V = 50 temperature, 22 °C; time, 10 s; [NB]/[E] feed ratio, 4 mol/mol; NB feedstock concentration, 0.57 mol/L; ^2^ measure of the electron-donating properties of phosphine ligands, as reported by Tolman [35]; ^3^ phosphine cone angle, as reported by Tolman [35]; ^4^ activity in g_pol_/mol_V_; ^5^ determined by ^13^C-NMR; ^6^ determined by SEC; ^7^ determined by differential scanning calorimeter (DSC).

## Supplementary Materials

The following are available online. Figure S1: ^13^C and ^1^H-NMR spectra of a selected PE. Figure S2: (a) DSC curve, and (b) XRD pattern of a selected PE.
