# Supplementary material for: Homo- and Copolymerization of Ethylene with Norbornene Catalyzed by Vanadium(III) Phosphine Complexes
Source: Molecules. 2019 May 31;24(11):2088. doi: 10.3390/molecules24112088 (PMC6600629; doi:10.3390/molecules24112088)
Supplement: Supplementary file 1 [file molecules-24-02088-s001.pdf]

Supplementary Materials

Homo- and Co-polymerization of Ethylene with Norbornene Catalyzed by Vanadium(III) Phosphine Complexes

Giorgia Zanchin[1], Alessia Gavezzoli[1,†], Fabio Bertini[1], Giovanni Ricci[1] and Giuseppe Leone [1,*]

[1] CNR-Istituto per lo Studio delle Macromolecole (ISMAC), via A. Corti 12, I-20133 Milano, Italy; giorgia.zanchin@ismac.cnr.it (G.Z.); alessia.gavezzoli@gmail.com (A.G.); fabio.bertini@ismac.cnr.it (F.B.); giovanni.ricci@ismac.cnr.it (G..R.)

* Correspondence: giuseppe.leone@ismac.cnr.it (G.L.)

† Present address: Loxeal srl, via G. Marconato 2, I-20811 Cesano Maderno (MB)

Table of Contents

Figure S1: ^{13}C and ^{1}H NMR spectra of a selected PE (entry 3 in Table 1).

Figure S2: (a) DSC curve, and (b) XRD pattern of a selected PE (entry 3 in Table 1).

Figure S1: ^{13}C and ^{1}H NMR spectra of a selected PE (entry 3 in Table 1).

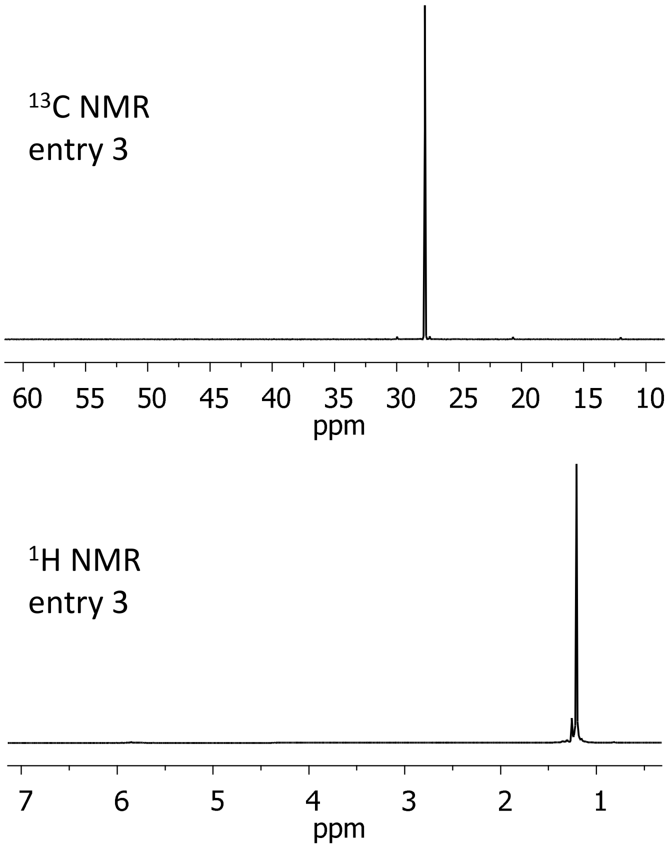

The intense signal at 27.73 ppm in the ^{13}C NMR spectrum, ascribed to methylene –CH_2– sequences is safely detected, whereas no signals due to isolated branches are detected, thus suggesting that the obtained PE is linear. In addition, no signals ascribed to olefinic protons, in the range from about 5.0 to 6.0 ppm in the ^{1}H NMR spectrum, are registered.

Figure S2: (a) DSC curve, and (b) XRD pattern of a selected PE (entry 3 in Table 1).

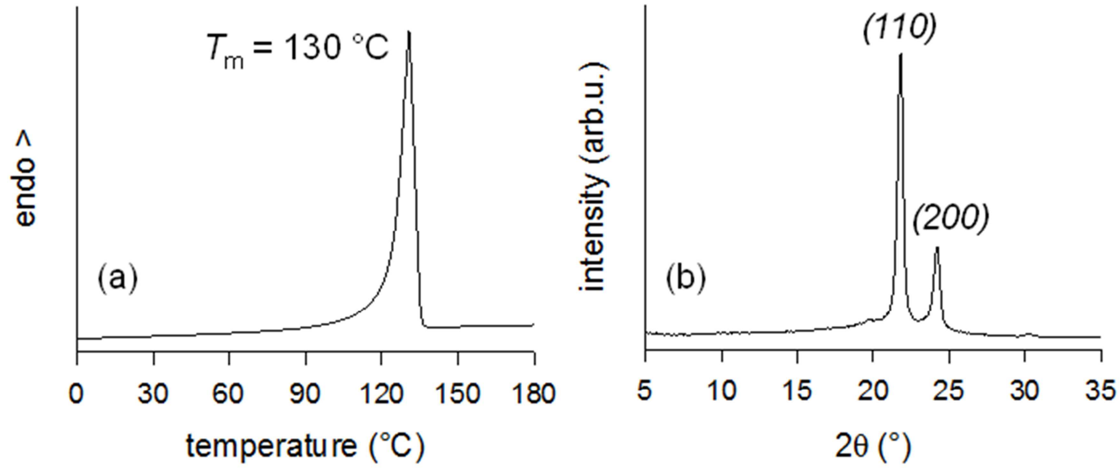

The DSC curve recorded during the second heating of the selected and representative sample 3 showed an endothermic peak at about 130 °C, consistent with the T_m of linear PEs, and in agreement with the X-Ray Powder Diffraction (XRD) profile (Figure S2b). Indeed, from the XRD pattern two sharp, well-defined, peaks at 21.73 and 24.18 °2θ, respectively, are registered, typical of semi-crystalline PEs (they represented the 110 and 200 planes of the orthorhombic PE cell, respectively).
